# Role of DDR1 in Regulating MMPs in External Root Resorption

**DOI:** 10.3390/ijms252212111

**Published:** 2024-11-11

**Authors:** Yuhan Wang, Bing Han, Hongyan Tian, Kaining Liu, Xiaoyan Wang

**Affiliations:** 1 Department of Cariology and Endodontology, Peking University School and Hospital of Stomatology & National Center for Stomatology & National Clinical Research Center for Oral Diseases & National Engineering Research Center of Oral Biomaterials and Digital Medical Devices, Beijing 100081, China; sakura_9@live.cn (Y.W.); hanbing822@126.com (B.H.); 2 First Clinical Division, Peking University School and Hospital of Stomatology & National Center for Stomatology & National Clinical Research Center for Oral Diseases & National Engineering Research Center of Oral Biomaterials and Digital Medical Devices, Beijing 100081, China; dentisthy@foxmail.com; 3 Department of Periodontology, Peking University School and Hospital of Stomatology & National Center for Stomatology & National Clinical Research Center for Oral Diseases & National Engineering Research Center of Oral Biomaterials and Digital Medical Devices, Beijing 100081, China

**Keywords:** discoidin domain receptor 1, matrix metalloproteinases, MAPK, periodontal ligament cells, PI3K/Akt, Smad2/3

## Abstract

Human periodontal ligament cells (hPDLCs) express matrix metalloproteinases (MMPs), a group of enzymes responsible for the destruction of most extracellular matrix proteins in dental tissues, especially MMP-1, MMP-2, and MMP-13. Exploring the regulatory mechanism of MMPs is crucial for understanding external root resorption (ERR), one of the most severe complications, along with substantial loss of dental tissue, induced by trauma, pulpal infection, tooth bleaching, and orthodontic treatment, etc. Discoidin domain receptor 1 (DDR1), a cell surface receptor binding to collagen, has the potential to regulate the expression of MMP-1, MMP-2, and MMP-13, but the mechanism remains unclear. Thus, the present study aimed to investigate the connection and underlying mechanism between MMP-1, MMP-2, MMP-13, and DDR1 in hPDLCs. Our post-replantation ERR model revealed that Mmp-1, Mmp-2, Mmp-13, and Ddr1 all increased in the sites of ERR. hPDLCs with DDR1 knockdown exhibited a substantial reduction in MMP-1, MMP-2, and MMP-13 expression. To further confirm the underlying mechanism, we conducted further in vitro experiments, including RNA sequencing, RNA interference, RT-qPCR, Western blotting, and ELISA. Based on our results, MMP-1 was positively regulated by the Smad2/3 and MEK-ERK1/2 pathways and negatively regulated by the PI3K-Akt pathway through CCN2. MMP-2 and MMP-13 were positively regulated by the Smad2/3 pathway. MMP-13 was positively regulated by the MEK-ERK1/2 and PI3K/Akt signaling pathways. Collectively, DDR1 is a potent regulator of MMP-1, MMP-2, and MMP-13 expression through the Smad2/3, MEK-ERK1/2, and PI3K/Akt signaling pathways. Clarifying the significance and underlying mechanism by which DDR1 is involved in ERR might bring the chances to hinder the pathogenic process of ERR, hence reducing its incidence rate.

## 1. Introduction

Matrix metalloproteinases (MMPs) are an essential family of zinc- and calcium-dependent endopeptidases playing a crucial role in the degradation of different extracellular matrix (ECM) and basement membrane components [1]. The dysregulation of MMPs is related to external root resorption (ERR), one of the most severe complications, along with substantial loss of dental tissue, induced by trauma, pulpal infection, tooth bleaching, and orthodontic treatment, etc. [2,3].

ERR is in large part caused by a pathologically excessive activity of MMPs [1]. Particularly, there is a strong link between the level of MMP-1, MMP-2, and MMP-13 expression and the severity of dental tissue destruction. MMP-1, the most commonly associated collagenase, has been found in sites with root resorption and recognized as a biomarker of inflammation [4,5]. MMP-2, one of the gelatinases, has received specific attention as an essential participant in the pathobiology of external root resorption [6]. MMP-13, the widest substrate selection among the interstitial collagenases, has been found to express in bovine root-resorbing tissues [7] and serves as a target for diagnosis and therapy [8].

In our earlier study, we investigated the positive impact of discoidin domain receptor 1 (DDR1) on the adhesive and migratory abilities of human periodontal ligament cells (hPDLCs) [9]. Moreover, hPDLCs have been verified to potentially contribute to the loss of dental tissues, which is largely caused by a substantial increase in the expression of MMP-1, MMP-2, and MMP-13 in hPDLCs [10,11,12]. This elucidates the possible involvement of DDR1 in ERR. As a collagen-specific receptor exhibiting tyrosine kinase activity [13], DDR1 is not only associated with more intracellular signaling cascades but also plays an indispensable role in the regulation of cell adhesion, migration, wound healing, and other functions [9,14]. It has been suggested that the upregulation of MMP-1, MMP-2, and MMP-13 expression may be related to DDR1 in various cells, such as smooth-muscle cells (SMCs) [15], ovarian cancer cells [16], and so on. However, the regulatory mechanisms behind DDR1 and these MMPs remain unclear as of now.

Given the importance of MMP-1, MMP-2, and MMP-13 in ERR, it is pivotal to investigate the correlation and regulatory mechanism behind MMP-1, MMP-2, MMP-13, and DDR1 in hPDLCs, particularly in pathological situations characterized by inflammatory stimulation. Hence, we propose the hypothesis that the expression of these MMPs in hPDLCs is related to DDR1, resulting in further exploration of the underlying molecular mechanism with the help of bioinformatics.

## 2. Results

### 2.1. Higher Levels of DDR1, MMP-1, MMP-2, and MMP-13 Expression Induced by ERR In Vivo

**Figure 1:**
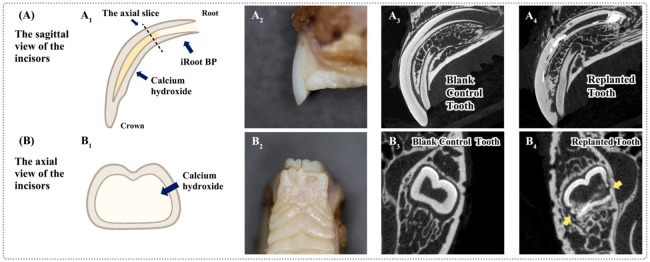
Establishment of external root resorption models in rabbits. (**A**,**B**) Sagittal and axial view of the incisors. (**A1**,**B1**) Schematic diagrams of the sagittal and axial slices. (**A2**,**B2**) Photos of the incisors as viewed from the sagittal and axial direction. (**A3**,**A4**) Micro-CT sagittal slices of replanted and control incisors, respectively. (**B3**,**B4**) Representative axial image of Micro-CT showing the control incisor and the replanted incisor. The region of ERR is indicated by the yellow arrows. (*n =* 8).

**Figure 2:**
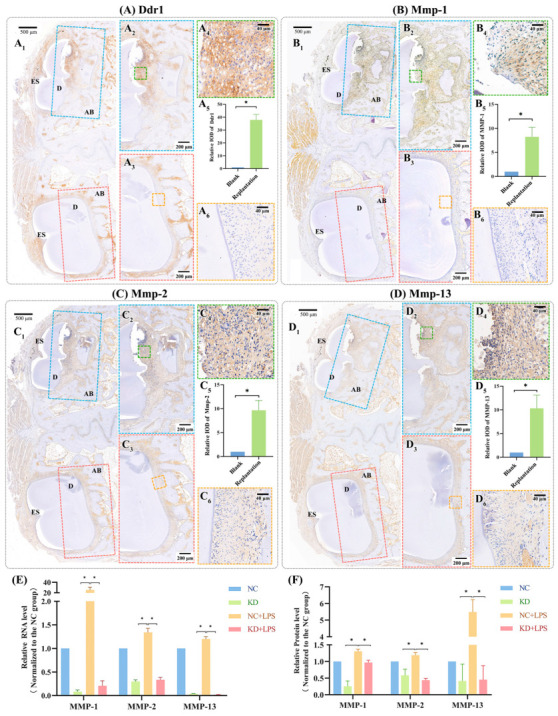
The effects of DDR1 on MMP-1, MMP-2, and MMP-13 expression in vivo and in vitro. (**A**–**D**) Representative IHC results of Ddr1, Mmp-1, Mmp-2, and Mmp-13 in the rabbit model. (**A1**,**B1**,**C1**,**D1**) Representative IHC images of periodontal tissues surrounding the blank control incisor (left) and the replanted incisor (right). (**A2**,**A4**,**B2**,**B4**,**C2**,**C4**,**D2**,**D4**) Magnified images of the replanted incisors. (**A3**,**A6**,**B3**,**B6**,**C3**,**C6**,**D3**,**D6**) Magnified images of the blank control incisors. (**A5**,**B5**,**C5**,**D5**) Quantified IHC data for Ddr1, Mmp-1, Mmp-2, and Mmp-13, respectively (all *p* < 0.05); (**E**) RT-qPCR results of MMP-1, MMP-2, and MMP-13 with and without DDR1 knockdown. (**F**) ELISA results of MMP-1, MMP-2, and MMP-13 with and without DDR1 knockdown. Protein concentration in ELISA: MMP-1 in the NC group: 316 pg/mL; MMP-2 in the NC group: 121 pg/mL; MMP-13 in the NC group: 147 pg/mL. ES: enamel space; D: dentin; AB: alveolar bone. Data are shown as mean ± SE. “*” marks a statistical difference between the corresponding groups (one-way ANOVA test, in vivo *n =* 8, in vitro *n =* 3).

Micro-computed tomography (Micro-CT) revealed significant ERR in the roots of replanted incisors, particularly in the central third sections of the roots (Figure 1A,B), indicating that in vivo ERR models were successfully formed. IHC staining revealed that the periodontal tissues in ERR locations expressed more Ddr1 (Figure 2A), Mmp-1 (Figure 2B), Mmp-2 (Figure 2C), and Mmp-13 (Figure 2D) than the control tooth, and the quantification findings demonstrated a significant difference (*p* < 0.05).

### 2.2. Lowered MMP-1, MMP-2, and MMP-13 Expression Caused by DDR1 Knockdown in hPDLCs

Over 80% knockdown efficiency for DDR1 has been confirmed in our previous study [9]. RT-qPCR (Figure 2E) and ELISA (Figure 2F) both showed that D1 knockdown induced remarkably decreases in MMP-1, MMP-2, and MMP-13 expression at the RNA and protein levels (*p* < 0.05). After being stimulated with *P. gingivalis* LPS (*Pg*-LPS), MMP-1, MMP-2, and MMP-13 exhibited an apparent increase, while the expression of the KD group was still significantly lower than that of the NC group (*p* < 0.05).

### 2.3. Differential Expression Analysis and KEGG Pathway Enrichment Analysis of RNA-seq

**Figure 3:**
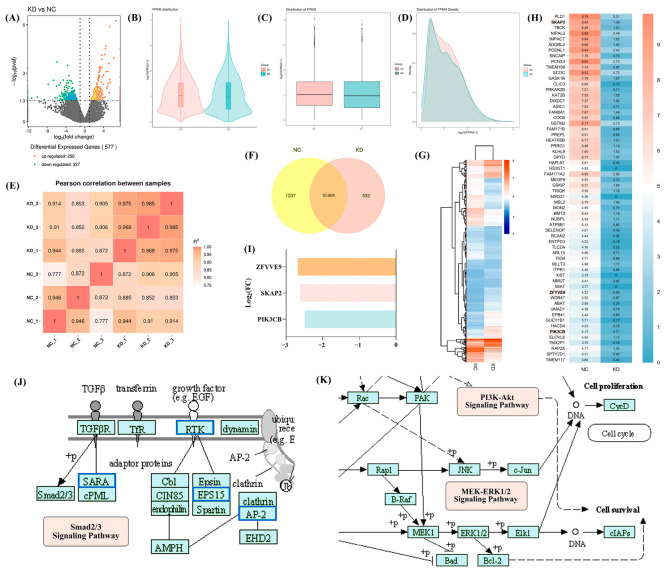
RNA-seq data of the NC group compared with the KD group. (**A**) Volcano plot of sequencing data. (**B**–**D**) Fragments per kilobase of exon per million fragments (FPKM) distribution of all RNA-seq data of the NC and KD groups. (**E**) Thermal diagram of the correlation coefficient among RNA-seq samples. (**F**) Venn diagram showing the overlap in the expression profiles of the NC group and the KD group. (**G**) Heatmap of RNA-seq data showing all 577 differentially expressed genes (*p* value&log_2_FC > 1). (**H**) Part of the heatmap that contains ZFYVE9 (SARA, encoding SMAD anchor for receptor activation), SKAP2 (encoding src kinase associated phosphoprotein 2), and PIK3CB (encoding PI3K) highlighted in the enlarged image. (**I**) Downregulated gene expression of ZFYVE9, SKAP2, and PI3KCB changes shown with a histogram. The *y*-axis is the log_2_FC of the RPKM ratio of the KD group to the NC group (all *p* < 0.05). (**J**,**K**) KEGG pathway enrichment analysis shows that the Smad2/3, MEK-ERK1/2, and PI3K/Akt signaling pathways are suppressed in the KD group. (*n =* 3).

A total of 577 differentially expressed genes were uncovered, among which 250 were upregulated and 327 were downregulated (Figure 3A). FPKM distribution was presented to quantify gene expression between the NC and KD groups (Figure 3B–D). A Venn diagram shows the overlap between the expression profiles of the NC and KD groups (Figure 3F). Among those, based on the differential expression analysis, ZFYVE9 (SARA, encoding SMAD anchor for receptor activation), SKAP2 (encoding src kinase associated phosphoprotein 2), and PIK3CB (encoding PI3K) were related to the Smad2/3, MEK-ERK1/2, and PI3K/Akt signaling pathways, respectively (Figure 3G–I). KEGG pathway enrichment analysis was performed for the 327 downregulated genes using the KEGG pathway database (http://www.kegg.jp/kegg/pathway.html, accessed from 1 June to 1 August 2022, in which the Smad2/3, MEK-ERK1/2, and PI3K/Akt signaling pathways were also identified (Figure 3J–K).

### 2.4. Decreased PI3K/Akt and Smad2/3 Expression Caused by DDR1 Knockdown in hPDLCs

**Figure 4:**
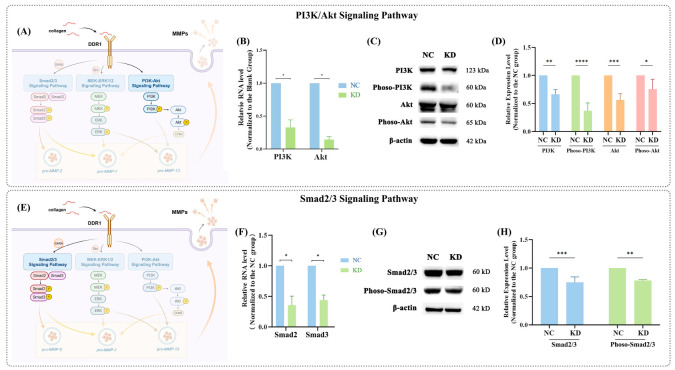
Effects of DDR1 knockdown on PI3K/Akt and Smad2/3 signaling pathways in hPDLCs. (**A**) The diagram implies that the PI3K/Akt signaling pathway might be one of the downstream pathways of DDR1. (**B**) According to the RT-qPCR results, after DDR1 knockdown, PI3K and Akt levels clearly descended (*p* < 0.05), which was consistent with the results of RNA-seq. (**C**,**D**) The Western blotting results of PI3K and Akt followed the same pattern as the RT-qPCR (*p* < 0.05). (**E**) The diagram shows that the Smad2/3 signaling pathway might be one of the downstream pathways of DDR1. (**F**) The RT-qPCR results reveal that DDR1 knockdown remarkedly reduced Smad2 and Smad3 expression (*p* < 0.05), which is indicated in the results of RNA-seq. (**G**,**H**) The Western blotting results of Smad2 and Smad3 were consistent with the RT-qPCR results (*p* < 0.05). Data are shown as mean ± SE. “*” marks a statistical difference between the corresponding groups (* *p* < 0.05; **, *p* < 0.01; ***, *p* < 0.001; ****, *p* < 0.0001) (one-way ANOVA test, *n =* 3).

According to the RT-qPCR data, the knockdown of DDR1 in hPDLCs resulted in an apparent decline in PI3K, Akt, Smad2, and Smad3 expression (*p* < 0.05) (Figure 4B,F). These results validated the RNA-seq data, as the relative expression levels of these genes were consistent in both the RT-qPCR and RNA-seq analyses. Western blotting analysis revealed that the downregulation of DDR1 led to a notable reduction in the protein expression of PI3K, phoso-PI3K, Akt, phoso-Akt (Figure 4C,D), Smad2/3, and phoso-Smad2/3 (Figure 3H and Figure 4G) (*p* < 0.05).

### 2.5. Effects of DDR1 on MMP-1 Expression via the Smad2/3, MEK-ERK1/2, and PI3K/Akt Signaling Pathways 

**Figure 5:**
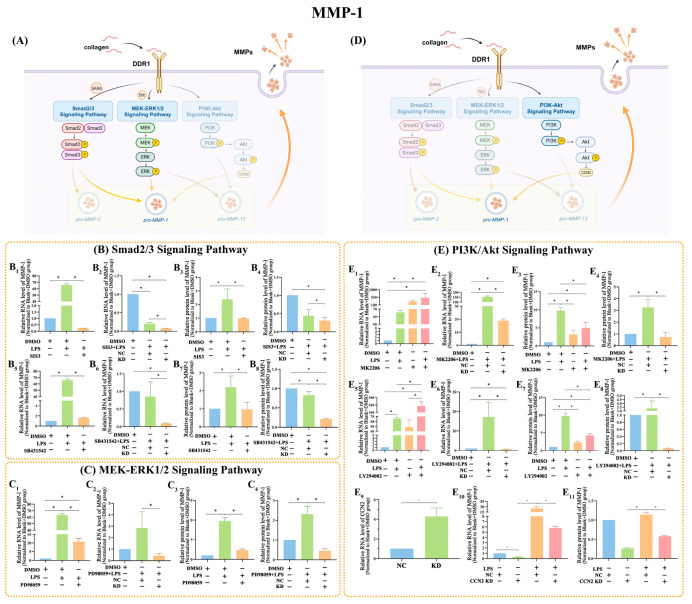
Effects of DDR1 knockdown on MMP-1 via the Smad2/3 and MEK-ERK1/2 signaling pathways. (**A**) The schematic diagram suggests the pattern by which DDR1 might regulate MMP-1 expression via the Smad2/3 and MEK-ERK1/2 pathways. (**B**) RT-qPCR and ELISA data indicate that the Smad2/3 signaling pathway has an impact on the expression of MMP-1 (*p* < 0.05). (**C**) The Smad2/3 signaling pathway influences the expression of MMP-1, as suggested by RT-qPCR and ELISA data (*p* < 0.05). (**D**) The pattern in the schematic diagram implies that DDR1 may control MMP-1 expression via the PI3K/Akt pathway by means of CCN2. (**E**) MMP-1 expression is influenced by the PI3K/Akt signaling pathway, as shown by the RT-qPCR and ELISA results (*p* < 0.05). Protein concentration in ELISA: MMP-1 in the Blank + DMSO group: 383 pg/mL; MMP-2 in the Blank + DMSO group: 258 pg/mL; MMP-13 in the Blank + DMSO group: 170 pg/mL. Data are shown as mean ± SE. “*” marked a statistical difference between the corresponding groups (one-way ANOVA test, *n =* 3).

The influence of DDR1 on MMP-1 expression was investigated using RT-qPCR and ELISA. The results obtained from RT-qPCR and ELISA shared identical patterns (Figure 5). With *Pg*-LPS added, cells exhibited a significantly increased level of MMP-1 (*p* < 0.05). Then, with the addition of the MEK inhibitors (PD98059) or the Smad2/3 inhibitors (SIS3 and SB431542), MMP-1 was significantly downregulated (*p* < 0.05) (Figure 5(B1,B3,B5,B7,C1,C3)). Moreover, MMP-1 expression further decreased when DDR1 siRNA was applied with a combination of the MEK inhibitor and the Smad2/3 inhibitors (*p* < 0.05) (Figure 5(B2,B4,B6,B8,C2,C4)).

Nevertheless, RT-qPCR and ELISA results both indicated that the suppression of the PI3K/Akt signaling pathway resulted in an unforeseen upregulation of MMP-1 expression (*p* < 0.05). By adding *Pg*-LPS, there was a further increased expression of MMP-1 (*p* < 0.05) (Figure 5(E1,E3,E5,E7)). However, when DDR1 was suppressed, this impact was partially reversed (*p* < 0.05) (Figure 5(E2,E4,E6,E8)). The upregulation of CCN2 was uncovered upon inhibition of DDR1 (*p* < 0.05) (Figure 5(E9)). In addition, the expression of MMP-1 at both the RNA and protein levels was considerably reduced when CCN2 was suppressed (*p* < 0.05) (Figure 4(E10,E11)). The knockdown efficiency of CCN2 is shown in Figure S1. The unprocessed blot scans are shown in Figure S2.

### 2.6. Effects of DDR1 on MMP-2 Expression via the Smad2/3 Signaling Pathway and MMP-13 Expression via the MEK-ERK1/2 and PI3K/Akt Signaling Pathways

**Figure 6:**
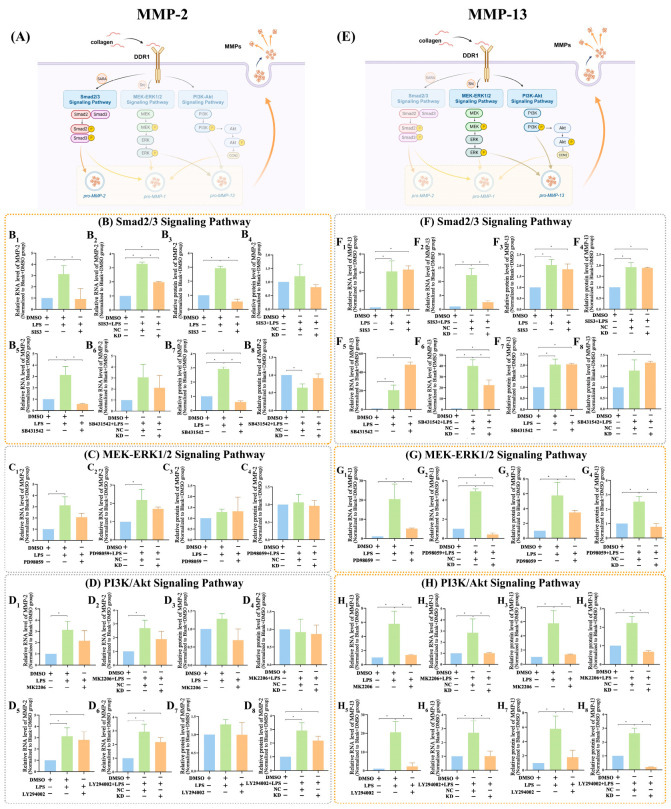
Effects of DDR1 knockdown on MMP-2 via the Smad2/3 signaling pathway and MMP-13 via the MEK-ERK1/2 and PI3K/Akt signaling pathways. (**A**) The schematic diagram demonstrates how DDR1 controls MMP-2 expression via the Smad2/3 signaling pathway other than the MEK-ERK1/2 and PI3K/Akt signaling pathways. (**B**) RT-qPCR and ELISA data revealed the regulation of MMP-2 expression via the Smad2/3 signaling pathway. (**C**) The MEK inhibitor (PD98059) did not inhibit the overexpression of MMP-2 caused by *Pg*-LPS, as indicated by the results from both RT-qPCR and ELISA (*p* > 0.05). (**D**) Both RT-qPCR and ELISA data showed no inhibition of *Pg*-LPS-induced MMP-2 overexpression by PI3K/Akt inhibitors (MK2206 and LY294002) (*p* > 0.05). (**E**) The schematic diagram illustrates that DDR1 affects MMP-13 expression through the MEK-ERK1/2 and PI3K-Akt signaling pathways instead of the Smad2/3 signaling pathway. (**F**) No statistically significant difference was observed between groups stimulated by *Pg*-LPS with and without Smad2/3 inhibitors (SIS3 and SB431542) according to RT-qPCR and ELISA results (*p* > 0.05). (**G**) According to RT-qPCR and ELISA results, the MEK-ERK1/2 signaling pathway influences MMP-13 expression (*p* < 0.05). (**H**) According to RT-qPCR and ELISA results, the PI3K/Akt signaling pathway affects MMP-13 expression (*p* < 0.05). Protein concentration in ELISA: MMP-1 in the Blank + DMSO group: 383 pg/mL; MMP-2 in the Blank + DMSO group: 258 pg/mL; MMP-13 in the Blank + DMSO group: 170 pg/mL. Data are shown as mean ± SE. “*” marks a statistical difference between the corresponding groups (one-way ANOVA test, *n =* 3).

The influence of DDR1 on the expression of MMP-2 and MMP-13 was further studied using RT-qPCR and ELISA, revealing consistent patterns (Figure 6). The addition of *Pg*-LPS resulted in a notable elevation in the expression of MMP-2 (*p* < 0.05). Subsequently, the inclusion of Smad2/3 inhibitors (SIS3 and SB431542) led to a notable decrease in MMP-2 expression (*p* < 0.05) (Figure 6(B1,B3,B5,B7)). Additionally, the expression of MMP-2 was significantly reduced when DDR1 siRNA was combined with the Smad2/3 inhibitor (SIS3) (*p* < 0.05) (Figure 6(B2,B4,B6,B8)). However, similar effects were not observed in the MEK-ERK1/2 and PI3K/Akt signaling pathways (*p* > 0.05) (Figure 6C,D).

As for MMP-13, the addition of *Pg*-LPS caused a significant increase in MMP-13 expression (*p* < 0.05). Using the MEK inhibitor (PD98059) and PI3K/Akt inhibitors (MK2206 and LY294002) resulted in a significant reduction in MMP-13 expression (*p* < 0.05) (Figure 6(G1,G3,H1,H3,H5,H7)). Furthermore, MMP-2 expression experienced a substantial decrease upon combining DDR1 siRNA with the MEK inhibitor (PD98059) or PI3K/Akt inhibitors (*p* < 0.05) (Figure 6(G2,G4,H2,H4,H6,H8)). Nevertheless, no comparable outcomes were detected in the Smad2/3 signaling pathways (*p* > 0.05) (Figure 6F).

## 3. Discussion

Our present study has substantially validated the hypothesis that “DDR1 has a crucial regulatory impact on the expression of MMP-1, MMP-2 and MMP-13” and that this regulation is partially mediated by the Smad2/3, MEK-ERK1/2, and PI3K/Akt pathways, which is in line with the previous studies [16,17,18]. The results also widen the scope of the relationship between DDR1 and MMPs expression, which contributes to elucidating the significance of DDR1 in ERR.

Considering the vital roles of MMPs [14], clarifying the DDR1-mediated regulation of MMP-1, MMP-2, and MMP-13 could help to understand the pathological process of ERR, potentially lowering the incidence rate. MMP-1, MMP-2, and MMP-13 have a particularly vital role in the degradation of collagens (collagen types Ⅰ, Ⅱ, Ⅲ, and Ⅳ). Collagen fibrils are initially cleaved by MMP-1 and MMP-13. Subsequently, MMP-2 could diffuse into the fibrils, unwind them, and further digest them [19]. MMP-1 and MMP-13 are secreted collagenases with a broad range of substrates that are crucial in the process of destroying interstitial collagens [7]. MMP-2, known as one of gelatinases, destroys collagen type Ⅰ and IV, the predominant components of the ECM, playing a role in the physiological and pathological remodeling of the ECM [6]. An upregulated expression of MMP-1, MMP-2, and MMP-13 is associated with different malignant pathologies [7,20], such as squamous-cell carcinomas of the mouth. So, it stands to reason that these MMPs would have some features and functions in common. As DDR1 has been verified to be significant in the development of dental and periodontal tissues [21] and the process of oral diseases [14], in the present study, we focused on the mechanisms controlling DDR1-mediated MMP-1, MMP-2, and MMP-13 expression in hPDLCs to demonstrate the role of DDR1 in ERR.

DDR1 is a unique class of receptor tyrosine kinases (RTKs), which could selectively bind to the GVMGFO motif in micro-environmental fibrillar collagens (collagen types Ⅰ, Ⅱ, Ⅲ, and Ⅳ). DDR1 is subsequently activated, triggering the downstream signaling pathways [22,23]. As is reported in the previous study, DDR1 may activate the ERK1/2, PI3K/Akt, JAK/STAT, and Rho-GTPase signaling pathways [24]. In osteoarthritis (OA) and rheumatoid arthritis (RA), where MMPs serve as important immune response factors as well, DDR1 is being explored as a potential therapeutic target [25]. Clarifying the relationship between DDR1 and MMPs, as well as its regulatory mechanism, is important for discovering the possible preventative and therapeutic strategies, as the involvement of MMPs in ERR was comparable to that of OA and RA.

Since DDR1 has been proved to be a potential target for preventing ERR, future medical research and development may employ this therapeutic approach by blocking DDR1. For example, DDR1-targeted extra-canal medicine might be utilized as a preventative measure against ERR after tooth avulsion or prior to intentional replantation/autotransplantation.

Previous studies have utilized several sorts of animals for ERR models, including mice, rats, and dogs [26]. The rabbit’s incisor was selected for our current study because of its sufficient size and potential for long-term breeding. In the present study, based on ERR models in rabbits, the expression levels of Mmp-1, Mmp-2, Mmp-13, and Ddr1 all exhibited a substantial rise in conjunction with the existence of ERR, uncovering certain associations between DDR1 and these MMPs in the pathogenic process of ERR.

In addition to in vivo ERR models, in vitro experiments also provided evidence of the correlation between DDR1 and the expression of MMP-1, MMP-2, and MMP-13 in hPDLCs. MMPs are deeply involved in dental and periodontal destruction [14]. Therefore, DDR1 is expected to play a regulatory role in oral diseases related with MMPs. Nevertheless, further data are still needed to demonstrate this correlation. Based on the findings from RT-qPCR and ELISA, it was noted that cells with DDR1 knockdown had notably reduced the levels of MMP-1, MMP-2, and MMP-13 expression. This observation aligns with the results of IHC staining in vivo, which suggests that DDR1 may function as a potential regulator of MMP-1, MMP-2, and MMP-13. Due to the low amount of MMPs secreted by hPDLCs without any treatment [27], *Pg*-LPS was applied to mimic the inflammatory situation in vivo, which induced cells to express more MMPs. LPS is an important component of Gram-negative bacteria and has a potent effect on immune response induction by a variety of cells [28]. Recognition of LPS by cells goes through Toll-like receptors (TLRs), a family of trans-membrane proteins, which are similar to the toll protein of Drosophila [29]. *Pg*-LPS was chosen due to its strong correlation with some oral diseases, e.g., periodontitis [30]. Differently from common LPSs, such as LPS derived from *Escherichia coli* (*E. coli*), *Pg*-LPS mainly activates TLR2 and is a relatively weak TLR4 agonist [31]. A previous study has documented that treatment with *Pg*-LPS resulted in an increase in the expression of MMPs [32], which is suitable for activating hPDLCs in our current work. The introduction of *Pg*-LPS resulted in a significant increase in the expression of MMP-1, MMP-2, and MMP-13, while a notable decrease was found after DDR1 knockdown. Collectively, DDR1 has a noticeable impact on the expression of MMP-1, MMP-2, and MMP-13 in vitro.

It is insufficient to confirm that DDR1 affects MMP-1 and MMP-13 both in vivo and in vitro; further research is needed to clarify its underlying mechanisms. RNA-seq data helped identify three potential downstream pathways of DDR1: the Smad2/3, MEK-ERK1/2, and PI3K/Akt signaling pathways. According to the RNA-seq data, ZFYVE9, also referred to as SARA, which encodes the SMAD anchor for receptor activation, showed a significant decrease, indicating that Smad2/3 might serve as a downstream part of DDR1. This finding was aligned with our results of RT-qPCR and Western blotting. Another study also demonstrated an association between the Smad2/3 signaling pathway and MMPs expression [33]. Additionally, according to the RNA-seq data, SKAP2 (encoding src kinase associated phosphoprotein 2) was obviously decreased in the differential expression analysis, which provided compelling evidence for DDR1-MAPK regulation [34]. Our prior research found that preventing DDR1 resulted in a noticeable decrease in the expression of MEK1/2, phoso-MEK1/2, ERK1/2, and phoso-ERK1/2, and that the role of DDR1 in cellular migration and adhesion may be also mediated through the MEK-ERK1/2 signaling pathway [9]. Similarly, the MEK-ERK1/2 pathway was found to not only be linked to DDR1 but also to play a critical role in regulating MMPs expression in SW1353 cells [35]. Finally, there was an apparent reduction in PIK3CB encoding PI3K according to the RNA-seq. The effect of DDR1 knockdown suggested that the PI3K/Akt signaling pathway might act as a downstream pathway of DDR1. The PI3K/Akt signaling pathway was reported to be the key pathway causing phenotype changes in associated cells to a more proinflammatory state [36], and it could interact with MMP expression in various kinds of cells, including LoVo cells [37], prostate cancer cells [38], etc. It might be worth studying the involvement of the Smad2/3, MEK-ERK1/2, and PI3K/Akt signaling pathways in the DDR1-mediated regulation of MMPs.

In the present study, MMP-1 was revealed to be regulated by DDR1 via the Smad2/3, MEK-ERK1/2, and PI3K/Akt signaling pathways, since the blockade of the Smad2/3 and MEK-ERK1/2 signaling pathways reversed *Pg*-LPS-induced increases in MMP-1. In comparison to applying the Smad2/3 inhibitors (SIS3 and SB431542) or MEK inhibitor (PD98059) alone, the reduction in MMP-1 was much greater when DDR1 knockdown was included, suggesting that there is more than one signaling pathway connecting DDR1 and MMP-1. Notably, with regard to the PI3K/Akt signaling pathway, the use of PI3K/Akt inhibitors (LY294002 or MK2206) intensified the upregulation of MMP-1. The application of DDR1 siRNA partially relieved this impact. A potential underlying factor could be cellular communication network 2 (CCN2), also known as connective tissue growth factor (CTGF), which is encoded by immediate early genes. CCN2 is a prototypical member of the CCN family of matricellular proteins [39]. CCN2 regulates numerous cellular processes, including adhesion, proliferation, apoptosis, and ECM protein synthesis [40]. Previous research indicated that the intimate control of CCN2 expression by the PI3K/Akt signaling pathway had an exceptional cell type-dependence [41]. Inhibition of this pathway generally increased the expression of CCN2 in SMCs, fibroblasts, epithelial cells, and other types of cells [41,42,43]. The downregulation of Akt signaling in human dermal fibroblasts led to the activation of CCN2, and CCN2 is required to be activated to promote the expression of MMP-1 after inhibiting Akt [44]. Moreover, CCN2 has been regarded as a key regulator of MMP-1 in multiple studies [45,46]. In our present study, the inhibition of DDR1 resulted in an increased expression of CCN2 and the change in MMP-1 followed the same trend in CCN2 expression. This result supports the involvement of CCN2 in the rise of MMP-1 expression observed in hPDLCs when PI3K/Akt is inhibited. The specific molecular mechanisms behind the activation of CCN2 through PI3K/Akt blockage are currently unclear and require further investigation. Furthermore, it appears that there could be additional mechanisms influencing the relationship between DDR1 and MMP-1. As reported in the previous study, MMP-1 also has the potential to cause Akt phosphorylation, which in turn acts on the PI3K/Akt signaling pathway [47,48]. Therefore, it is feasible that the LPS-induced MMP-1 expression accelerate the phosphorylation of Akt, raising the level of MMP-1. With various signaling pathways regulating MMP-1 expression through DDR1, the expression of MMP-1 tends in the same direction as DDR1.

DDR1 appears to regulate MMP-2 via the Smad2/3 signaling pathway as opposed to the MEK-ERK1/2 and PI3K/Akt pathways in hPDLCs. Blocking the Smad2/3 signaling pathway prevented the increase in MMP-2 produced by *Pg*-LPS. However, there was no difference observed between the groups with and without MEK and PI3K/Akt inhibitors, suggesting that these pathways might have no effects on the expression of MMP-2. According to previous findings, the expression of MMP-2 could be greatly affected by the Smad2/3 signaling pathway in human vein endothelial cells [33] and HT-29 cells [49], which was also verified in our present study in hPDLCs. Additionally, DDR1 is observed to control MMP-13 expression via the MEK-ERK1/2 and PI3K/Akt signaling pathways, rather than the Smad2/3 pathway in hPDLCs. The MEK inhibitor PD98059 and the PI3K/Akt inhibitors MK2206 and LY294002 effectively blocked the *Pg*-LPS-induced the upregulation of MMP-13. Nevertheless, no such effects were noted upon inhibition of the Smad2/3 signaling pathway, which revealed that these pathways might not have any impact on the expression of MMP-13. Previous research has shown that the inhibition of the PI3K/Akt pathway decreased the production of MMP-13 in SW1353 cells [50]. Furthermore, it has also been confirmed that the MEK-ERK1/2 signaling pathway regulates MMP-13 expression in chondrocytes [51]. Lastly, however, compared to using the inhibitors alone, the reduction in MMP-2 or MMP-13 was significantly greater when DDR1 was knocked down, indicating that there might be multiple signaling pathways connecting MMP-2, MMP-13 and DDR1, which merits further research.

Moreover, notably, the identification of the Smad2/3 signaling pathway supports other reports that Smad expression and phosphorylation is associated with PI3K and ERK [52,53,54]. However, more attention should be paid to transforming growth factor β (TGF-β). TGF-β is a multifunctional cytokine involved in the regulation of cell proliferation, differentiation, and survival of many cells [55]. It also plays a crucial role in signal propagation by phosphorylating Smad [56]. Particularly, TGF-β may promote immunosuppression by preventing activated macrophages from producing proinflammatory cytokines [57], and immunosuppression is necessary for the poor differentiation responses or inappropriate physiological responses associated with osteogenic and odontogenic cells [58]. Therefore, since the expression of TGF-β was not examined in the present study, there is a possibility that immunosuppression occurs in ERR, intensifying DDR1-mediated loss of dental hard tissue. Further research is still warranted. Additionally, while DDR1 was bound to and activated by collagen, this study did not investigate the interaction between DDR1 and collagen in the context of ERR. The previous study noted that distinct cleavages of collagen may have distinct effects on DDR1 activation [59]. Therefore, more investigation is required to determine whether similar effects play a role in the process of ERR. Although we focus on endogenous MMPs secreted by cells, the present study did not cover exogenous MMPs released by oral pathogens. More research is necessary since oral pathogens might promote the level of MMPs directly or indirectly [60].

In summary, our data support the hypothesis that DDR1 may be largely involved in regulating the expression of MMP-1, MMP-2, and MMP-13, which would lead to ERR. Moreover, our results demonstrate that the regulation of MMP-1, MMP-2, and MMP-13 through DDR1 does not follow the same pattern, which may indicate that the level of connection between DDR1 and different MMP varies. Further investigation is required to verify the in-depth regulatory mechanisms of DDR1 on MMP-1, MMP-2, and MMP-13.

## 4. Materials and Methods

### 4.1. External Root Resorption Models in Rabbits

All animal procedures were in accordance with the ethical guidelines and regulations of the Institutional Animal Care and Use Committee at Peking University Health Science Center (LA2022002). Three healthy male New Zealand white rabbits, 6 to 8 weeks of age and 2.4 to 2.7 kg in weight, were used. Tooth replantation procedures were as follows (Figure S3): under anesthesia, the central incisors of male rabbits were extracted. Then, pulp was removed and the roots were rinsed with and placed in saline. Calcium hydroxide was injected into the root canal as intercanal medication and iroot BP (Innovative Bioceramix Inc, Vancouver, Canada) was used as the root-end filling material and replanted into the sockets. The entire procedure took ten minutes or less. Finally, flowable resin was used to secure the transplanted and control central incisors. Then, all the rabbits were kept at the animal facility with free access to water and crushed food in order to avoid any occlusal trauma. A postoperative antibiotic (gentamicin) was administered intramuscularly at 20,000 units per day for 3 days. The rabbits were sacrificed eight weeks after replantation. Incisors were randomly assigned to experimental and control groups. The replanted incisor was employed as the experimental tooth (*n =* 8) while the control tooth was thought to be the other incisor (*n =* 8). Samples measuring approximately 5 cm × 2 cm, including incisors, periodontal tissues, and alveolar bone, were selected for additional experiments. Blinding was carried out as follows: the researchers in charge of data collection and analysis did not know the group division at all. All rabbits used in this study were used for the data analysis.

### 4.2. Micro-Computed Tomography (Micro-CT)

After rabbits were sacrificed, samples comprising the incisors and surrounding tissues were obtained and scanned on a micro-CT machine (Skyscan 1276; Bruker micro-CT, Kontich, Belgium) with a 20.4 μm voxel size. The X-ray tube was operated at a voltage of 100 kV and a current of 200 µA.

### 4.3. Immunohistochemistry (IHC)

The rabbit samples were fixed in 4% paraformaldehyde, decalcified (with 0.5 mM EDTA at pH 8.0), dehydrated, embedded in paraffin, and then sectioned at 4 µm thickness. Following these steps, after deparaffinization, rehydration, antigen unmasking, and endogenous peroxidase blocking, sections were blocked in 5% BSA with 0.1% Triton X-100 in PBS. Sections were incubated overnight at 4 °C with primary antibodies. The antibodies are as follows: anti-DDR1 (Santa Cruz Biotechnology, Dallas, TX, USA), anti-MMP-1 (BioLegend Inc., San Diego, CA, USA), anti-MMP-2 (Servicebio, Wuhan, China), and anti-MMP-13 (Servicebio, Wuhan, China). Sections were then stained using an immunohistochemistry kit (Servicebio, Wuhan, China). At last, sections were observed under a microscope (Olympus, Tokyo, Japan) and photographed after being counterstained with hematoxylin, dried, and mounted. Data of three samples were measured using Fiji (https://imagej.net/Fiji, accessed from 1 January to 1 June 2022).

### 4.4. High-Throughput RNA Sequencing

RNA was isolated from the NC and KD groups, and then the RNA was subjected to high-throughput RNA sequencing (RNA-seq) using the Illumina high-throughput sequencing platform (Illumina, San Diego, CA, USA) supplied by Allwegene Technology Co., Ltd. (Beijing, China). Raw data (raw reads) in the fastq format were firstly processed through in-house perl scripts. In this step, clean data (clean reads) were obtained by removing reads containing adapters, reads containing ploy-N, and low-quality reads from raw data. At the same time, Q20, Q30, GC-content, and the sequence duplication level of the clean data were calculated. All the downstream analyses were based on clean data with high quality. The RNA levels were estimated using fragments per kilobase of exon per million fragments (FPKM) mapped. Differential expression analysis was performed using DESeq R packages (version 4.4.1). A *p* value and log_2_FC (fold change) >1 was set as a threshold of differentially expressed genes (DEGs). KEGG pathway analysis was performed using KOBAS software (version 3.0) [61] to examine the statistical enrichment of DEGs.

### 4.5. Cell Culture and Grouping

Extracted third molars were obtained from nine volunteers with healthy periodontal tissues. Cells from the different donors were not mixed. This study was approved by the institutional review board of Peking University School and Hospital of Stomatology (PKUSSIRB-2011007). The primary cells were cultured according to our previous protocols [9,62,63,64]. Periodontal tissues were collected from the central section of the extracted tooth root by gently curetting and mincing. T25 flasks (Corning, Inc., Corning, NY, USA) were used for hPDLCs isolated from the periodontal tissue blocks. Then, hPDLCs were cultured in Dulbecco’s modified Eagle’s medium (Gibco, Waltham, MA, USA) with 1% penicillin–streptomycin and 10% fetal bovine serum (Kang Yuan Biology, Tianjin, China). The hPDLCs were placed in an incubator containing 5% CO_2_ at a temperature of 37 °C.

The cells were digested using 0.25% trypsin and 0.02% EDTA when hPDLCs reached 80% confluence. Cells derived from passage 4 were used in subsequent experimental procedures and the cells were further segregated into different groups, as detailed in Table 1 and Table 2.

### 4.6. RNA Interference

Small RNA interference was used to knock down DDR1 and CCN2. After reaching 80% confluence in 6-well plates (1 × 10^5^ cells/well), the hPDLCs were then transfected with siRNA (GenePharma, Shanghai, China). Ten nanomolar siRNAa were pre-complexed with jetPRIME transfection reagent (Polyplus-Transfection, Strasbourg, France) at room temperature for 10 min before adding to hPDLCs. Then, for RT-qPCR, the cells were grown in 5% CO_2_ at 37 °C for 48 h, and for Western blotting and ELISA, they were cultured for 72 h. The siRNA sequences for DDR1 were 5′-CCACCAACUUCAGCAGCUUTT-3′ (sense) and 5′-AAGCUGCUGAAGUUGGUGGTT-3′ (antisense). The siRNA sequences for CCN2 were 5′-GCACCAGCAUGAAGACAUATT-3′ (sense) and 5′-UAUGUCUUCAUGCUGGUGCTT-3′ (antisense). Cells with DDR1 knockdown were labeled as the KD group, as shown in Table 1, while cells with CCN2 knockdown were denoted as the CCN2 KD group.

### 4.7. RNA Extraction and RT-qPCR

RNA was obtained using TRIzol reagent (Invitrogen, Carlsbad, MA, USA) following the manufacturer’s protocol. The quality and integrity were assessed by NanoDrop spectrophotometer (Thermo Scientific, Waltham, DE, USA). Reverse transcription was performed using a PrimeScript RT Master Mix kit (Takara Bio, Shiga, Japan), and 500 ng of RNA was used to obtain cDNA. RT-qPCR was then performed using SYBR Green (Vazyme Biotech Co., Ltd., Nanjing, China) with a real-time thermocycler (Applied Biosystems, Waltham, MA, USA). The primers sequences are displayed in Table 3.

### 4.8. ELISA

The protein concentration of MMP-1, MMP-2, and MMP-13 in culture supernatants was measured using ELISA kits (Proteintech, Wuhan, China) following the manufacturer’s protocols. The standard curves of MMP-1 and MMP-13 were prepared from 31.25 to 2000 pg/mL, and 100 µL/well were added in duplicate to the microplate. The samples were diluted at least fourfold in the same diluent as the standards, and 100 µL of these were added to the plates and incubated at 37 °C for 2 h. Then, the detection antibody (1×, 100 µL/well) was added to the microplate and incubated at 37 °C for 1 h. The microplate was developed using 100 µL of streptavidin–horseradish peroxidase (HRP, 1×, 100 µL/well) followed by 100 µL of tetramethylbenzidine substrate (TMB). The reaction was stopped using 100 µL/well of stop solution and measured at 450 nm with a plate reader (BioTek, Winooski, VT, USA). The sample concentrations were back-calculated using the standard curve fitted with four parameters. The results were normalized to the respective controls.

### 4.9. Western Blotting

Western blotting was conducted according to the protocol described previously [9]. Protein concentration was measured using a BCA protein assay kit (Solarbio, Beijing, China). Equal amounts of protein were separated by electrophoresis on 12% sodium dodecyl sulfate–polyacrylamide gels; then, the gels were electroblotted onto polyvinylidene fluoride (PVDF) membranes (Bio-Rad, Hercules, CA, USA). The membranes were blocked for 30 min in 5% non-fat milk. After blocking, the membranes were incubated overnight with primary antibodies at 4 °C. The primary antibodies (ABclonal, Wuhan, China) used were as follows: anti-Smad2/3, anti-phoso-Smad2/3, anti-PI3K, anti-phoso-PI3K, anti-Akt, anti-phoso-Akt, and anti-β-actin. After washing with PBST, the membranes were incubated with secondary antibodies (ZSGB-BIO, Beijing, China) and detected by peroxidase-conjugated secondary antibodies using an enhanced chemiluminescence (ECL) system (NCM Biotech, Suzhou, China). The blots were scanned using a Western blot detection system. Band intensity was analyzed using Fiji (https://imagej.net/Fiji, accessed from 1 January to 1 June 2023). Protein expression was normalized to β-actin.

### 4.10. Statistical Analysis

The data were analyzed using SPSS Statistics 25.0 software (SPSS, Inc., Chicago, IL, USA). The Shapiro–Wilk test was used to demonstrate the distribution of variants. If the data were normally distributed, t test and one-way analysis of variance (ANOVA) were used, and the data were presented as mean ± standard error of the mean. If the data were not normally distributed, the Kruskal–Wallis test was used, and the data were presented as median and interquartile range. A *p* value < 0.05 indicated a statistically significant difference.

## 5. Conclusions

Conclusively, DDR1 may have a role in regulating the expression of MMP-1, MMP-2, and MMP-13, contributing to ERR. This regulation is likely achieved through the Smad2/3, MEK-ERK1/2, and PI3K/Akt signaling pathways. MMP-1 was positively regulated by the Smad2/3 and MEK-ERK1/2 pathways and negatively regulated by the PI3K/Akt pathway through CCN2. MMP-2 was positively regulated by the Smad2/3 pathway. MMP-13 was positively regulated by the MEK-ERK1/2 and PI3K/Akt signaling pathways. With the elucidation of the importance and underlying mechanism of DDR1 involved in ERR, DDR1 can be further applied as a target for the prevention and treatment of ERR.

## Figures and Tables

**Table 1:** The groups for DDR1 knockdown.

Group	Treatment
Blank	Cells without any treatment
Negative control (NC)	Cells transfected with NC siRNA (siRNA nonhomologous to any known gene sequence)
Knockdown (KD)	Cells transfected with DDR1 siRNA
Negative control + LPS (NC + LPS)	Cells transfected with NC siRNA and stimulated with LPS derived from *P. gingivalis* (*Pg*-LPS; 10 μg/mL; Sigma-Aldrich, St. Louis, MO, USA) for 24 h
Knockdown + LPS (KD + LPS)	Cells transfected with DDR1 siRNA and stimulated with *Pg*-LPS for 24 h

**Table 2:** The groups for MEK-ERK1/2, PI3K/Akt, and Smad2/3 inhibition.

Group	Treatment
Blank + DMSO	Pre-treated with DMSO
Blank + DMSO + LPS	Pre-treated with DMSO and treated with *Pg*-LPS
PD98059 + LPS	Pre-treated with PD98059 at the concentration of 20 µM and treated with *Pg*-LPS
PD98059 + LPS + NC	Pre-treated with PD98059 and treated with NC siRNA and *Pg*-LPS
PD98059 + LPS + KD	Pre-treated with PD98059 and treated with DDR1 siRNA and *Pg*-LPS
MK2206	Pre-treated with Akt inhibitor (MK2206) at a concentration of 10 µM for 24 h
MK2206 + LPS	Pre-treated with MK2206 and treated with *Pg*-LPS
MK2206 + LPS + NC	Pre-treated with MK2206 and treated with NC siRNA and *Pg*-LPS
MK2206 + LPS + KD	Pre-treated with MK2206 and treated with DDR1 siRNA and *Pg*-LPS
LY294002	Pre-treated with PI3K inhibitor (LY294002) at a concentration of 12 µM for 24 h
LY294002 + LPS	Pre-treated with LY294002 and treated with *Pg*-LPS
LY294002 + LPS + NC	Pre-treated with LY294002 and treated with NC siRNA and *Pg*-LPS
LY294002 + LPS + KD	Pre-treated with LY294002 and treated with DDR1 siRNA and *Pg*-LPS
SIS3 + LPS	Pre-treated with SIS3 at a concentration of 10 μM and treated with *Pg*-LPS
SIS3 + LPS + NC	Pre-treated with SIS3 and treated with NC siRNA and *Pg*-LPS
SIS3 + LPS + KD	Pre-treated with SIS3 and treated with DDR1 siRNA and *Pg*-LPS
SB431542 + LPS	Pre-treated with SB431542 at a concentration of 10 μM and treated with *Pg*-LPS
SB431542 + LPS + NC	Pre-treated with SB431542 and treated with NC siRNA and *Pg*-LPS
SB431542 + LPS + KD	Pre-treated with SB431542 and treated with DDR1 siRNA and *Pg*-LPS

**Table 3:** Sequences of primers.

Gene	Forward Primer (5′-3′)	Reverse Primer (5′-3′)	Size of Amplified Products
*DDR1*	AAGGGACATTTTGATCCTGCC	CCTTGGGAAACACCGACCC	181 bp
*MMP-1*	AAAATTACACGCCAGATTTGCC	GGTGTGACATTACTCCAGAGTTG	82 bp
*MMP-2*	TACAGGATCATTGGCTACACACC	GGTCACATCGCTCCAGACT	90 bp
*MMP-13*	ACTGAGAGGCTCCGAGAAATG	GAACCCCGCATCTTGGCTT	103 bp
*PI3K*	TATTTGGACTTTGCGACAAGACT	TCGAACGTACTGGTCTGGATAG	190 bp
*Akt*	AGCGACGTGGCTATTGTGAAG	GCCATCATTCTTGAGGAGGAAGT	96 bp
*CCN2*	AAAAGTGCATCCGTACTCCCA	CCGTCGGTACATACTCCACAG	109 bp
*Smad2*	CCGACACACCGAGATCCTAAC	GAGGTGGCGTTTCTGGAATATAA	125 bp
*Smad3*	GCGTGCGGCTCTACTACATC	GCACATTCGGGTCAACTGGTA	233 bp
*GAPDH*	GAAGGTGAAGGTCGGAGTC	GAAGATGGTGATGGGATTTC	226 bp

## Data Availability

The data that support the findings of this study are available from the corresponding author upon reasonable request. The raw sequence data reported in this paper have been deposited in the Genome Sequence Archive [65] in the National Genomics Data Center [66], China National Center for Bioinformation/Beijing Institute of Genomics, Chinese Academy of Sciences (GSA: HRA006831), and they are publicly accessible at https://ngdc.cncb.ac.cn/gsa, accessed on 29 May 2024.

## Supplementary Materials

The following supporting information can be downloaded at https://www.mdpi.com/article/10.3390/ijms252212111/s1.
