# Supplementary material for: Role of DDR1 in Regulating MMPs in External Root Resorption
Source: Int J Mol Sci. 2024 Nov 11;25(22):12111. doi: 10.3390/ijms252212111 (PMC11594854; doi:10.3390/ijms252212111)
Supplement: Supplementary file 1 [file ijms-25-12111-s001.zip › ijms-3283646-supplementary.pdf]

Supplemental Material

Supplemental Figures

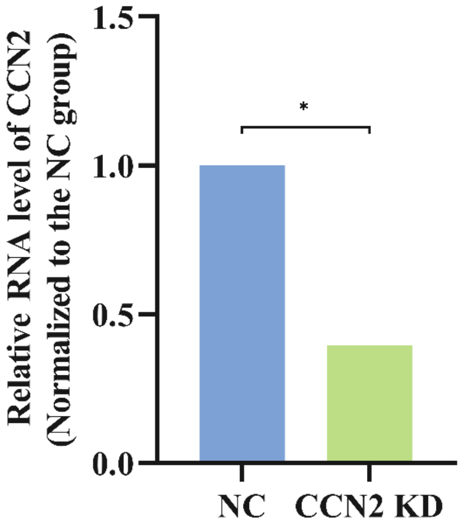

Figure S1. The interference efficiency of CCN2. The knockdown efficiency of CCN2 was verified compared to the NC group (P < 0.05).

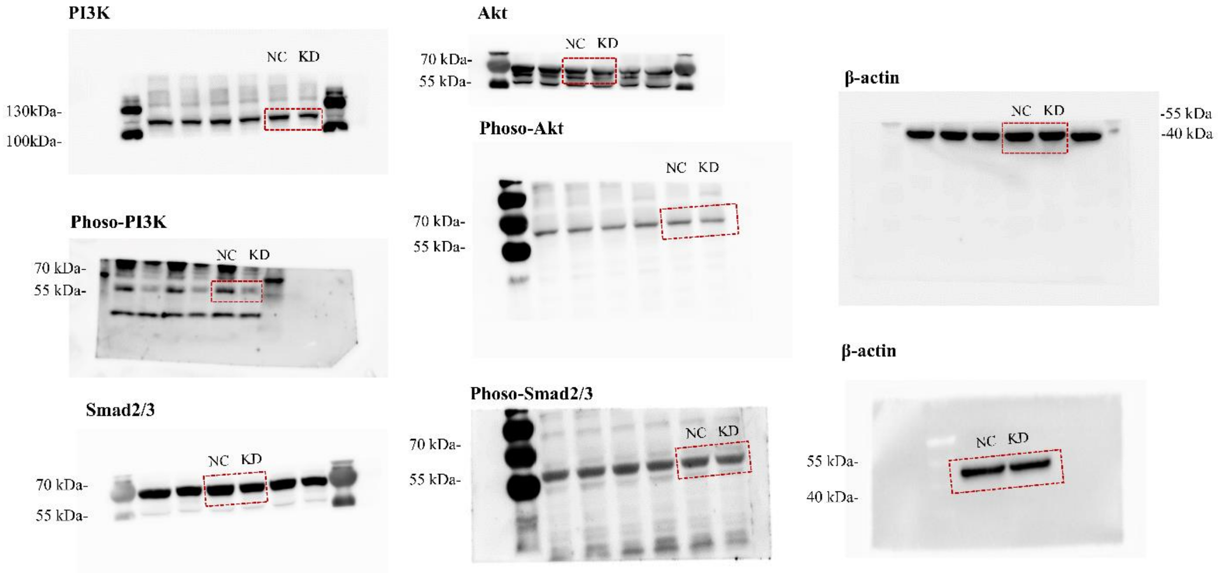

Figure S2. The unprocessed blot scans of western blotting. The red boxes represent the target proteins.

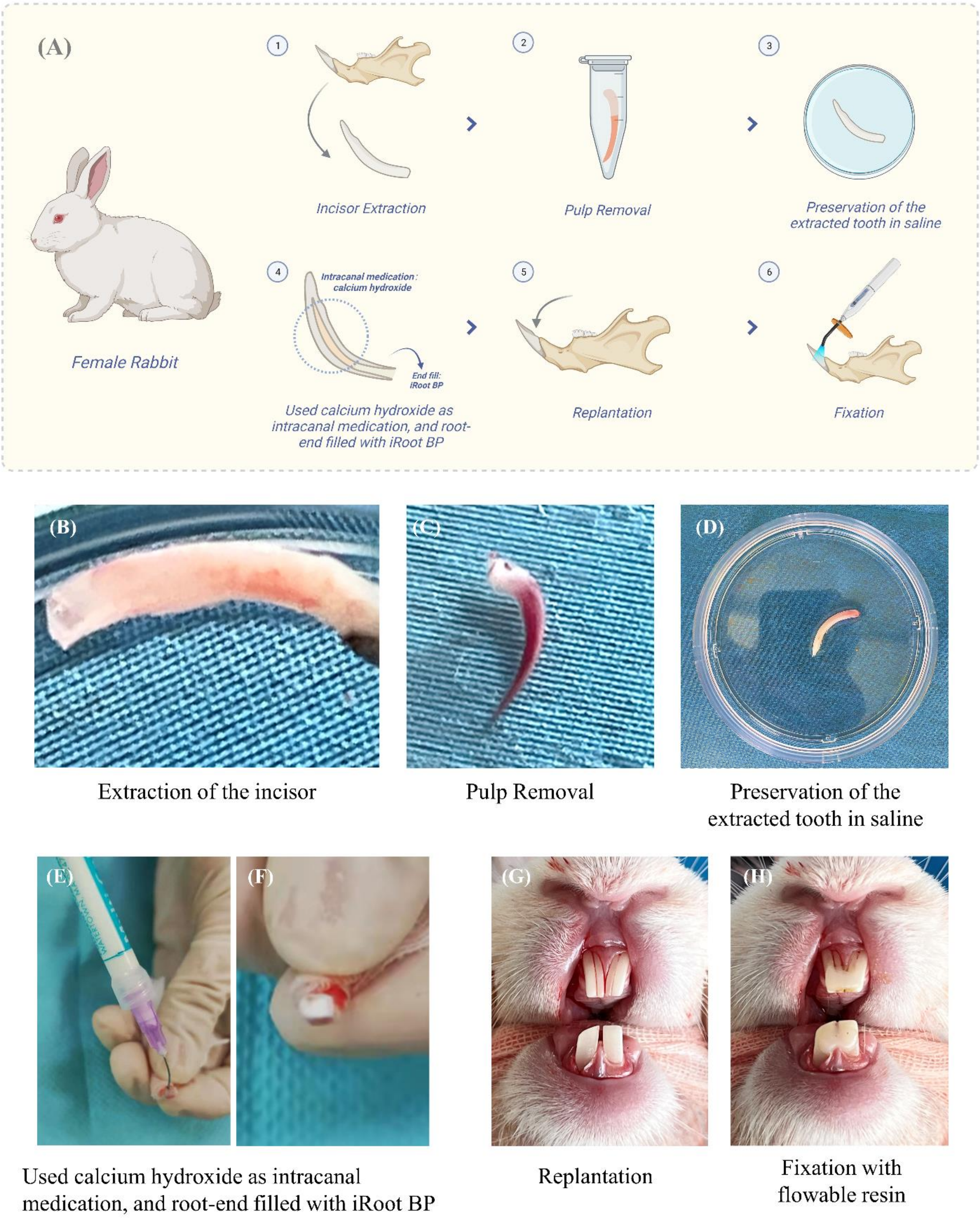

Figure S3. External root resorption models through tooth replantation in rabbits. (A) The schematic drawing illustrating the animal experimental procedure. (B) The central incisor was extracted. (C) The pulp was completely removed. (D) The

extracted incisor was preserved in saline before replantation. (E-F) The calcium hydroxide was used as intracanal medication and the root-end fill was used with iRoot BP. (G) The extracted incisor was replanted as before. (H) The incisors were fixed with the light-cured flowable resin.
